# Association between Physical Activity and Cardiovascular Risk in Chinese Youth Independent of Age and Pubertal Stage

**DOI:** 10.1186/1471-2458-10-303

**Published:** 2010-06-03

**Authors:** Alice PS Kong, Kai-Chow Choi, Albert MC Li, Stanley SC Hui, Michael HM Chan, YK Wing, Ronald CW Ma, Christopher WK Lam, Joseph TF Lau, Wing Yee So, Gary TC Ko, Juliana CN Chan

**Affiliations:** 1 Department of Medicine and Therapeutics, The Chinese University of Hong Kong, Prince of Wales Hospital, Shatin, Hong Kong SAR, China; 2 Li Ka Shing Institute of Health Sciences, The Chinese University of Hong Kong, Prince of Wales Hospital, Shatin, Hong Kong SAR, China; 3 The Nethersole School of Nursing, The Chinese University of Hong Kong, Shatin, Hong Kong SAR, China; 4 Department of Paediatrics, The Chinese University of Hong Kong, Prince of Wales Hospital, Shatin, Hong Kong SAR, China; 5 Department of Sports Science and Physical Education, The Chinese University of Hong Kong, Shatin, Hong Kong SAR, China; 6 Department of Chemical Pathology, The Chinese University of Hong Kong, Prince of Wales Hospital, Shatin, Hong Kong SAR, China; 7 Department of Psychiatry, The Chinese University of Hong Kong, Prince of Wales Hospital, Shatin, Hong Kong SAR, China; 8 School of Public Health and Primary Care, The Chinese University of Hong Kong, Prince of Wales Hospital, Shatin, Hong Kong SAR, China

## Abstract

**Background:**

Childhood and adolescence are critical periods of habit formation with substantial tracking of lifestyle and cardiovascular risk into adulthood. There are various guidelines on recommended levels of physical activity in youth of school-age. Despite the epidemic of obesity and diabetes in China, there is a paucity of data in this regard in Chinese youth. We examined the association of self-reported level of physical activity and cardiovascular risk in Hong Kong Chinese youth of school-age.

**Methods:**

This was a cross-sectional study conducted in 2007-8 in a school setting with 2119 Hong Kong Chinese youth aged 6-20 years. Physical activity level was assessed using a validated questionnaire, CUHK-PARCY (The Chinese University of Hong Kong: Physical Activity Rating for Children and Youth). A summary risk score comprising of waist circumference, blood pressure, fasting plasma glucose and lipids was constructed to quantify cardiovascular risk.

**Results:**

In this cohort, 21.5% reported high level of physical activity with boys being more active than girls (32.1% versus 14.1%, p < 0.001). Regression analysis showed physical activity level, sex and pubertal stage were independently associated with cardiovascular risk score.

**Conclusion:**

Self-reported level of physical activity is associated with cardiovascular risk factors in Chinese youth after adjusting for sex and pubertal stage.

## Background

Regular physical activity prevents myocardial infarction, cardiovascular events and premature mortality [1,2]. The beneficial effects of lifestyle modification, including regular exercise [3] on prevention of obesity, diabetes and hypertension are now confirmed [4,5] Obesity is a major risk factor of cardiovascular diseases [6]. With increasing childhood obesity, there is increasingly early onset of atherosclerosis [7]. Thus, there is an urgent need to emphasize healthy lifestyle and regular exercise especially in the youth to curb this public health problem.

Childhood and adolescence are critical periods of habit formation with substantial tracking of lifestyle and cardiovascular risk factors into adulthood [8,9]. In a systematic review, school-age youth are recommended to participate in at least 60 minutes of moderate to vigorous physical activity on a daily basis [10]. In a recent Chinese survey comprising more than 40,000 adults over the age of 20, the prevalence of diabetes was 9.7% with obesity as a major risk factor. Furthermore, the young to middle-aged group had the most rapid increase in diabetes prevalence over the last decades [11]. Despite this impending epidemic of young onset diabetes and associated co-morbidities, there is a paucity of data on physical activity level and its associations with cardiovascular risk factors in Chinese youth to increase awareness and promote lifestyle changes [12]. Hong Kong is one of the most affluent cities in China with 2-3% of the youth having metabolic syndrome [13]. In this study, we examined the level of physical activity in these young subjects and its associations with cardiovascular risk factors.

## Methods

### Participants

This was a cross-sectional study conducted from February 2007 to April 2008. A complete list of all primary and secondary schools in Hong Kong was obtained from the Education Department of Hong Kong. Hong Kong comprises of 18 districts. Cluster sampling method was used and had previously been described in the Hong Kong Growth Survey [14]. In brief, one primary school and one secondary school were randomly selected from each of these 18 districts in Hong Kong. The randomization was based on a computer-generated coding system. Among these 18 primary and 18 secondary schools selected in the Hong Kong Growth Survey, 5 primary schools and 6 secondary schools were randomly selected. All participants were healthy volunteers with Chinese ethnicity. This study was approved by the Clinical Research Ethics Committee of The Chinese University of Hong Kong. Informed written consents were obtained from all participants and their parents or guardians.

Anthropometric indexes were measured and fasting blood samples were obtained from each participant during the field study by a team of trained research nurses and research assistants. We used the same set of apparatus and methodology as described in our previous school children study [15]. Waist circumference (WC) was measured, to the nearest 0.1 cm, midway between the lowest rib and the superior border of the iliac crest in the midaxillary line. Body weight (measured to the nearest 0.1 kg by Tanita physician digital scale, model number TBF 410, Tanita Corp., Tokyo, Japan), height (measured to the nearest 0.1 cm using a portable standiometer) and blood pressure (BP) were measured twice after at least 5 minutes of rest with an average BP calculated using a validated electronic device (Omron, model number T5, Omron Healthcare Inc., Tokyo, Japan). Body mass index (BMI) was calculated from body weight (kg) divided by the square of body height (m^2^). All participants were invited to complete 2 validated questionnaires to assess their physical activity levels [16] and pubertal staging [17].

The CUHK-PARCY (The Chinese University of Hong Kong: Physical Activity Rating for Children and Youth) is a 1-item activity rating modified from the Jackson Activity Coding [18] and the Godin-Shephard Activity Questionnaire modified for Adolescents [19]. The CUHK-PARCY adopts a 11-point physical score (0-10) to grade levels of physical activity ranging from no exercise at all (0) to vigorous exercise on most days (10), taking into consideration the frequency, duration and intensity of the activity concerned. Secondary school students were asked to complete the questionnaire by themselves and primary school children were assisted by parents or guardians in answering the questionnaire. The English and Chinese versions of CUHK-PARCY were attached as additional files 1 and 2 respectively.

After an overnight fast of at least 8 hours, blood samples were collected for measurement of fasting plasma glucose and lipid profile including total cholesterol (TC), triglyceride (TG) and high-density lipoprotein cholesterol (HDL-C) levels. Low-density lipoprotein cholesterol (LDL-C) level was calculated using the Friedewald's formula for TG <4.5 mmol/l [20].

### Laboratory assays

All blood samples were kept in ice at 0°C before they were transported back to our laboratory for further processing. Blood samples including fasting plasma glucose and lipid profile were assayed within 6 hours after collection and additional aliquots of serum for other assays were stored at -70°C. Glucose (hexokinase method), TC (enzymatic method), TG (enzymatic method without glycerol blanking) and HDL-C (direct method using PEG-modified enzymes and dextran sulfate) were measured on a Roche Modular Analytics system (Roche Diagnostics GmbH, Mannheim, Germany) using standard reagent kits supplied by the manufacturer of the analyzer. The precision performance of these assays was within the manufacturer's specifications.

### Statistical Analysis

A summary risk score, based on The European Youth Heart Study [21], was constructed to quantify cardiovascular risk for the population sample of school children in Hong Kong. Specifically, the score consists of five components: WC, TG, fasting plasma glucose, TC/HDL-C ratio and systolic BP. The risk score was computed by summing up the following parameters:

(1) z-score of sex specific age-adjusted WC

(2) z-score of sex specific age-adjusted TG

(3) z-score of sex specific age-adjusted fasting plasma glucose

(4) z-score of sex specific age-adjusted TC/HDL-C ratio

(5) z-score of sex specific age and height adjusted systolic BP.

Each of the component variables of the risk score was regressed with age (and height as well for systolic BP) for boys and girls separately. The standardized residuals were retained to represent the z-score of age-adjusted values for each component variable.

Continuous variables with skewed and normal like distribution were presented as medians (inter-quartile ranges) and means (standard deviations) respectively. Categorical data were presented as counts and percentages. TG and TC/HDL-C ratio were natural log-transformed to correct their skewness before analysis. Overweight and obesity were defined by BMI greater than Hong Kong local age and sex specific 85th and 95th percentile respectively [22]. The local percentile standards were based on a population survey in Hong Kong [23]. Association between the cardiovascular risk score and level of physical activity with adjustment for demographic variables was assessed using regression models. The ordinal variable 'level of physical activity' was entered into analysis using the approach of Walter et al [24] to identify the most significant cutoff threshold. Physical activity and demographic variables (sex, age and pubertal stage) were entered into regression analyses with forward selection. Significant variables in any previous models were retained. All statistical analyses were performed using SPSS 16.0 (SPSS Inc., Chicago, IL). All statistical tests were two-sided and a p-value < 0.05 was considered statistically significant.

## Results

A total of 2119 Hong Kong Chinese school children aged 6-20 years consented to participate in this study (804 primary school students and 1315 secondary school students). Among them, 17 were excluded due to active medical or psychiatric illnesses with or without concomitant medications. All clinical information was based on history taking and questionnaire screening by research nurses or blood test abnormality. One primary school student was incidentally detected to have markedly elevated white cell counts and subsequently confirmed to have chronic myeloid leukemia by bone marrow examination. Among 2102 eligible subjects, 1882 completed the physical activity questionnaire. There were 115 boys and 105 girls who did not complete the physical activity questionnaire and were excluded from the analysis. The mean age of non-responders were 11.2 ± 2.6 and 11.2 ± 2.9 years in boys and girls respectively which were significantly younger than responders. Furthermore, since the majority of them were at pre- (Tanner stage 1, 50.6% boys and 33.7% girls) or early pubertal stage (Tanner stage 2, 25.9% boys and 37.3% girls), their anthropometric and clinical characteristics were expected to be different and thus excluded from the analysis.

Table 1 shows the demographic and clinical characteristics of the study cohort. The mean age of boys and girls was 13.2 ± 3.3 years and 13.8 ± 3.3 years respectively. 9% boys and 5.6% girls were obese. About one-third of the study cohort was in late or post-pubertal stage (31.6% boys and 39.9% girls were in Tanner stage 4; 3.4% boys and 9.9% girls were in Tanner stage 5) (Table 1). One fifth (21.5%) of the study cohort reported high level, and 78.5% reported moderate or low level of physical activity. More boys reported high level of physical activity than girls (32.1% versus 14.1%, p < 0.001).

**Table 1:** Demographic and clinical characteristics of 1882 Hong Kong Chinese school children aged 6-20 years who completed the physical activity survey

Characteristics	Boys (n = 780)	Girls (n = 1102)
Age (years)	13.2 (3.3)	13.8 (3.3)
Weight (kg)	47.7 (15.0)	44.2 (11.5)
Height (cm)	155.6 (17.0)	151.7 (12.6)
Body Mass Index (kg/m^2^)	19.2 (3.4)	18.9 (3.1)
Waist circumference (cm)	66.9 (9.8)	64.3 (7.9)
Systolic blood pressure (mmHg)	114.0 (12.2)	107.3 (9.7)
Diastolic blood pressure (mmHg)	67.0 (8.7)	67.0 (7.9)
HDL-C (mmol/L)	1.6 (0.3)	1.6 (0.3)
LDL-C (mmol/L)	2.1 (0.6)	2.2 (0.6)
TC/HDL-C ratio^†^	2.6 (2.3 - 3.0)	2.6 (2.3 - 2.9)
Triglyceride (mmol/L)^**†**^	0.7 (0.6 - 1.0)	0.7 (0.6 - 1.0)
Fasting plasma glucose (mmol/L)	4.8 (0.4)	4.7 (0.3)
Obesity status^ψ^		
Normal	598 (76.7%)	918 (83.3%)
Overweight	112 (14.4%)	122 (11.1%)
Obese	70 (9.0%)	62 (5.6%)
Puberty (tanner stage)^ψ^		
1	178 (24.0%)	163 (15.1%)
2	156 (21.1%)	122 (11.3%)
3	148 (20.0%)	255 (23.7%)
4	234 (31.6%)	429 (39.9%)
5	25 (3.4%)	107 (9.9%)
Physical activity score^ψ^		
0 - 2 (Low)	117 (15.0%)	306 (27.8%)
3 - 6 (Moderate)	413 (52.9%)	641 (58.2%)
7 - 10 (High)	250 (32.1%)	155 (14.1%)

Data marked with ^† ^were presented as medians (interquartile ranges) and with ^ψ ^as frequencies (%), all others were presented as means (standard deviations).

Table 2 shows the clinical characteristics and cardiovascular risk factor scores of the study cohort stratified by level of physical activity. Subjects with high physical activity were more likely to be boys, less obese, had lower Z score of TC/HDL-C ratio and less likely to be in the pre- pubertal stage. In the final regression model, female and high physical activity level were negatively associated, while puberty was positively associated with cardiovascular risk score (Table 3).

**Table 2:** Characteristics of clinical parameters and cardiovascular risk score of the study cohort stratified by level of physical activity (low to moderate versus high).

	Level of physical activity^#^		
	
Characteristics	Low to Moderate (n = 1477)	High (n = 405)	p-value
Sex^ψ^			
Male	530 (35.9%)	250 (61.7%)	<0.001
Female	947 (64.1%)	155 (38.3%)	
Age (years)	13.5 (3.4)	13.7 (2.9)	0.313
Age and sex adjusted values (in z-score):			
waist circumference	0.03 (1.02)	-0.04 (0.93)	0.208
systolic blood pressure(also height-adjusted)	0.00 (1.00)	-0.02 (0.99)	0.667
TC/HDL-C ratio	0.03 (1.00)	-0.10 (0.98)	0.030
triglyceride	0.02 (1.00)	-0.07 (0.96)	0.087
fasting plasma glucose	0.00 (1.00)	0.02 (1.01)	0.654
Cardiovascular Score *	0.07 (2.82)	-0.22 (2.77)	0.068
Obesity status^ψ^			
Normal	1180 (79.9%)	336 (83.0%)	0.009
Overweight	188 (12.7%)	46 (11.4%)	
Obese	109 (7.4%)	23 (5.7%)	
Puberty (tanner stage)^ψ^			
1	287 (20.1%)	54 (13.8%)	0.002
2 - 3	508 (35.6%)	173 (44.2%)	
4 - 5	631 (44.2%)	164 (41.9%)	

Data marked with ^ψ ^were presented as frequencies (%), all others were presented as means (standard deviations).

^# ^The cutoff threshold was identified using the approach of Walter et al [24]

Overweight and obesity were defined respectively by body mass index > 85^th ^and 95^th ^percentiles (age and sex specific based on a local population survey [23]).

* Cardiovascular Score = Sum of components' z score: components of cardiovascular risk score include z-score of sex-specific, age-adjusted waist circumference, systolic blood pressure (also height-adjusted), fasting plasma glucose, triglyceride and total cholesterol (TC)/high-density lipoprotein cholesterol (HDL-C) ratio.

**Table 3:** Results of regression analyses of the cardiovascular risk score in Hong Kong Chinese youth.

	Coefficient	SE	p-value
Model 1^†^			
PA level: Low to moderate (ref)			
High	-0.290	0.159	0.068
			
Model 2^#^			
PA level: Low to moderate (ref)			
High	-0.414	0.163	0.011
Sex: Male (ref)			
Female	-0.471	0.136	0.001
			
Model 3^#^			
PA level: Low to moderate (ref)			
High	-0.410	0.163	0.012
Sex: Male (ref)			
Female	-0.465	0.136	0.001
Age	-0.011	0.020	0.579
			
Model 4^#^			
PA level: Low to moderate (ref)			
High	-0.455	0.165	0.006
Sex: Male (ref)			
Female	-0.564	0.139	<0.001
Puberty (tanner stage):			
1 (ref)			
2 - 3	0.411	0.190	0.031
4 - 5	0.545	0.187	0.004

^† ^The baseline regression model included only level of physical activity (PA) and intercept. Regression coefficient of the intercept term was not shown.

^# ^Forward regression modeling was used for entering demographic variables: sex, age and puberty. Demographic variables significant in any pervious models were retained. Regression coefficient of the intercept term was not shown.

SE, standard error of the coefficients; PA, physical activity; ref, reference group of the categorical variable analyzed by creating dummy variables

* Cardiovascular Score = Sum of components' z score: components of cardiovascular risk score include z-score of sex-specific, age-adjusted waist circumference, systolic blood pressure (also height-adjusted), fasting plasma glucose, triglyceride and total cholesterol (TC)/high-density lipoprotein cholesterol (HDL-C) ratio.

## Discussion

In this survey, we observed that Chinese boys were more active than girls while physical activity and female were associated with low cardiovascular risk. After adjusting for known effects of puberty on cardiovascular risk factors, increased physical activity remained independently associated with low risk score.

The beneficial effect of regular exercise is possibly mediated through its direct effect on the circulatory system, improvement of sensitivity to insulin and adrenalin, increased non-insulin-dependent glucose uptake, improved oxidative enzymes in fat and carbohydrate metabolism [9]. In adults, regular and accumulated physical activities have been shown to prevent premature death and other adverse health outcomes [25]. In a large-scale, population-based cohort of Japanese adults aged 45-74 years, subjects in the top quartile of daily total physical activity level had lower risk of all-cause mortality than those in the lowest quartile [2].

In the youth population, association between physical activity and cardiovascular risk remains relatively unexplored. In support of our findings, in the United States, high physical activity level (≥60^th ^percentile in the study cohort) is associated with low prevalence of metabolic syndrome in adolescents [26]. In addition, Chinese girls had lower cardiovascular risk than boys after adjusting for pubertal staging and physical activity. In this regard, there are major gender differences in lifestyle, levels of physical activity, energy expenditure, body fat composition and hormonal levels. These differences may contribute to gender-related predisposition to health problems such as higher risk of hypertension, dyslipidemia and central obesity in men and higher risk of psychological problems such as depression and insomnia in women [27]. In a review consisting of 38 prospective cohort studies from Medline search (1966-2000) conducted primarily in the United States and Europe, women adhering to guidelines for physical activity had lower all-cause mortality rate than men with similar degrees of physical activity [28], suggesting that the protective effects of physical activity may be confounded by gender.

In this survey, only 21.5% of Chinese youth reported high level of exercise, a finding similar to the European survey in school children [29], with boys being more active than girls. These gender differences had also been reported in a Swedish study of 993 high school students aged 16-19 years surveyed by questionnaires [29]. In another study involving 2185 European children aged 9 and 15 years in whom physical activity levels were measured by accelerometers, boys were more active than girls [30]. In the European Youth Heart Study which included 1732 school children from Denmark, Estonia and Portugal (aged 9 year-old and 15 year-old), the authors reported graded and negative association between clustering of cardiovascular risk factors and physical activity. Based on these data, the authors recommended at least 90 minutes of daily physical activity in youth to prevent cardiovascular risk [21]. However, in this study, only pre- and post-pubertal school children were included and did not address the potential effects of puberty on cardiovascular risk. In our survey, onset of puberty was associated with increased cardiovascular risk score probably due to hormonal changes and their effects on insulin resistance.

There are ongoing debates on the optimal frequency, duration and intensity of physical activity [1,2]. Based on a systematic review of over 850 publications, school-aged youth were recommended to engage in at least 60 minutes of daily physical activity of moderate to vigorous intensity [10]. Here, most guidelines recommend high level or vigorous physical activity in young and active individuals while moderate activity appears to confer most benefits in the majority of people especially amongst the older and less active individuals [1,2]. In support of this notion, some studies have shown that while there was no association between mortality and light or low level of physical activity (<4 METs), vigorous or high level of physical activity (≥6 METs) was associated with increased mortality rate [31].

The first guideline for physical activity in the youth was published in 1988 by the American College of Sports Medicine which recommended that children and adolescents should have 20-30 minutes of vigorous exercise daily [32]. In 2007, the Regional Office for Europe of the World Health Organization (WHO) recommended children and young people to have at least 60 minutes of moderate-intensity physical activity daily. The guideline also highlighted that moderate-intensity physical activity at least twice weekly would enhance and maintain muscular strength, flexibility and bone health [33].

Several limitations of this study need to be addressed. First, due to budget restrictions, we used questionnaire rather than objective assessment tools like accelerometer to measure physical activity level. Nonetheless, questionnaires are widely accepted in large-scale surveys with reasonable reliability for analysis. For the same reason, we only analyzed fasting plasma glucose and did not measure insulin level to estimate insulin resistance as reported by other authors [21]. Second, we used cardiovascular risk factors to derive the risk score and long term prospective studies will be needed to confirm the validity of these risk scores (and associated physical activity level) in predicting cardiovascular diseases. Third, it might be difficult for children to recall physical activity levels over a long period of 12 months. However, such recall bias is likely to weaken rather than strengthen the risk associations.

## Conclusion

In this survey, we documented for the first time, the independent associations between self-reported physical activity on cardiovascular risk in Chinese youth after adjustment for sex and pubertal stage. Given the modifiable nature of physical activity and sedentary lifestyle of our young individuals, public health education, policies and prevention strategies are needed to promote physical activity in the youth to curb the rising tide of young-onset cardiovascular disease.

## Competing interests

The authors declare that they have no competing interests.

## Authors' contributions

APSK, AMCL prepared the proposal and supervised the study. APSK and KCC contributed to interpreting the results and writing the final manuscript and were responsible for data management and analysis. JTFL provided advice in data analysis. SSCH, YKW, RCWM, WYS and JCNC made substantial contributions to the conception of the study and revising the manuscript. MHMC and CWKL supervised the laboratory procedures. GTCK contributed to interpreting the results and revising the manuscript. All authors read and approved the final manuscript.

## Pre-publication history

The pre-publication history for this paper can be accessed here:

http://www.biomedcentral.com/1471-2458/10/303/prepub

## Supplementary Material

Additional file 1 **CUHK-PARCY (English version)**. A 1-item questionnaire to assess the physical activity levels of the participants of the study. Click here for file

Additional file 2 **CUHK-PARCY (Chinese version)**. A 1-item questionnaire to assess the physical activity levels of the participants of the study. Click here for file
