# Supplementary material for: Association between Physical Activity and Cardiovascular Risk in Chinese Youth Independent of Age and Pubertal Stage
Source: BMC Public Health. 2010 Jun 3;10:303. doi: 10.1186/1471-2458-10-303 (PMC2893096; doi:10.1186/1471-2458-10-303)
Supplement: Additional file 1 — CUHK-PARCY (English version). A 1-item questionnaire to assess the physical activity levels of the participants of the study. [file 1471-2458-10-303-S1.DOC]

**CUHK-PARCY** (The Chinese University of Hong Kong: Physical Activity Rating for Children and Youth) (English version)
